# Plant Community Responses to Simultaneous Changes in Temperature, Nitrogen Availability, and Invasion

**DOI:** 10.1371/journal.pone.0123715

**Published:** 2015-04-16

**Authors:** Elise S. Gornish, Thomas E. Miller

**Affiliations:** 1 Department of Plant Sciences, University of California Davis, Davis, California, United States of America; 2 Department of Biological Sciences, Florida State University, Tallahassee, Florida, United States of America; North Carolina State University, UNITED STATES

## Abstract

**Background:**

Increasing rates of change in climate have been observed across the planet and have contributed to the ongoing range shifts observed for many species. Although ecologists are now using a variety of approaches to study how much and through what mechanisms increasing temperature and nutrient pollution may influence the invasions inherent in range shifts, accurate predictions are still lacking.

**Methods and Results:**

In this study, we conducted a factorial experiment, simultaneously manipulating warming, nitrogen addition and introduction of *Pityopsis aspera*, to determine how range-shifting species affect a plant community. We quantified the resident community using ordination scores, then used structural equation modeling to examine hypotheses related to how plants respond to a network of experimental treatments and environmental variables. Variation in soil pH explained plant community response to nitrogen addition in the absence of invasion. However, in the presence of invasion, the direct effect of nitrogen on the community was negligible and soil moisture was important for explaining nitrogen effects. We did not find effects of warming on the native plant community in the absence of invasion. In the presence of invasion, however, warming had negative effects on functional richness directly and invasion and herbivory explained the overall positive effect of warming on the plant community.

**Conclusions and Significance:**

This work highlights the variation in the biotic and abiotic factors responsible for explaining independent and collective climate change effects over a short time scale. Future work should consider the complex and non-additive relationships among factors of climate change and invasion in order to capture more ecologically relevant features of our changing environment.

## Introduction

One of the most dramatic effects of climate change has been the worldwide increase in the frequency and extent of movement by species into new places [1, 2]. Multiple climate change drivers, including rising atmospheric temperatures and nutrient pollution, have been shown to affect the invasion of new species by changing or by reducing limiting resources [3, 4]. For example, climate change can shift resource availability directly [5], making it easier for a colonizer to establish a population by taking advantage of new conditions. Alternatively, climate-induced extinction of natives can change resource availability indirectly by affecting the extant native community [6], making a habitat more or less conducive to invasion. A subsequent ecological cascade often follows these invasions [7, 8, 9, 10].

Recent studies have attempted to quantify the combined effects of invasion and other climate change factors in order to capture a more realistic understanding of biological response to a changing environment. Many of these studies have found that simultaneous factors of climate change have non-additive effects (e.g., [11, 12, 13, 14], but see [15, 16]). In fact, a recent review documented a negative relationship between the number of climate change factors and overall effect size in plants [17]. Collectively, these studies suggest that we might not be able to make realistic predictions of the comprehensive effects of climate change based on experiments that simply record the response of ecological communities to a single changing environmental condition. Non-additive effects also hint at complex relationships among factors of climate change, ecological response, and underlying mechanisms that can explain these effects. For example, Verheyen and colleagues [18] found that both temperature and nitrogen deposition negatively affected the regeneration success of a threatened shrub, but the factors driving these relationships differed. Temperature was responsible for a reduction in seed number per cone, while nitrogen deposition enhanced cone predation [18]. In order to develop a better understanding of the relationship between climate change and biological response, process based studies that attempt to identify the biotic and abiotic factors actually explaining these responses must become a more common component of field experiment.

Very few studies have investigated the combined effects of two of the most important components of climate change—increasing temperature and nitrogen deposition- and newly established species on native communities [19], despite expectation of an interaction [20, 21]. Complex dynamics are expected to occur because ecological response to all three of these factors can be similar, despite differences in the variables that ultimately explain these effects. For example, nutrient cycling can be modified as a result of nitrogen deposition, increasing temperature and the presence of invasion (e.g., [1, 22]). The mechanism driving these changes, however can differ between nitrogen deposition and warming (i.e. changes in litter; [23, 24]), and invasion (i.e., [1]).

A more complete understanding of the relationships between climate change elements and invasive species requires the manipulation of all factors separately and in combination, while quantifying effects directly on resources, as well as in changes of the resident plant community. In this work, we asked, over a short time scale (1) how does increased temperature and nitrogen deposition influence a resident plant community and (2) how can invasion influence the plant community response to elevated temperature and nitrogen? We hypothesized warming and elevated nitrogen deposition to increase the abundance of invasive species found in our plant communities. This is because extinctions and range shifts often occur in response to increased temperature and nitrogen, which subsequently reduce community stability [25]. And, communities in transition may be more vulnerable to the effects of invasion [26]. We also expected invasion to enhance elevated temperature and nitrogen [27, 28] because these factors can modify habitats in similar ways, subsequently increasing overall effects when occurring at the same time. Further, we hypothesized that plant diversity would be reduced by the simultaneous effects of warming, nitrogen addition, and invasion compared to any of these independently [29, 30]. However, we expected that the factors explaining community response to combinations of invasion and warming, and invasion and nitrogen deposition would be different than factors mediating community response to warming and nitrogen deposition alone.

## Materials and Methods

This study was conducted in a 1.6 hectare old-field at Tall Timbers Research Station, just south of the Florida-Georgia border. Tall Timbers provided a permit to access the experimental site. This area was last used for agriculture ca. 150 years ago, and prior to the start of the experiment, the field had been disked annually. The field harbors a diverse community, dominated by native grasses and legumes and did not involve endangered or protected species. The soil type is a slightly acidic sandy loam. Precipitation at the site averages 100 cm per year, and the average annual temperature is 20°C.

The two year experiment was initiated in 2011, using a randomized block design with three main factors, each with two levels: nitrogen (N addition and no N addition), warming (warming and no warming), and invader (invader present and absent), for a total of 8 treatments. To minimize leaching of nitrogen between plots, the plots were arranged in a split-plot design, with N treatments arranged together within blocks with warming and invader treatments randomized within these blocks. Each block of treatments was replicated 5 times, for a total of 40 plots [31]. Each plot was 4 m^2^, but measurements were only collected from the center 1 m^2^ to minimize edge effects. Plots were separated by 1 m. This experiment was nested within a larger study, so data from some plots will not be discussed here (e.g. nitrogen + warming).

We implemented the nitrogen treatments by applying sodium nitrate (NaNO_3_) at the soil surface in the amount of 4 g/m² per year, based on projected dry + wet nitrogen deposition rates for northern Florida [32]. Six applications of equal amounts of NaNO_3_ were applied during the growing season (May—October) in 2011 and 2012. The warming treatment was applied with open-top hexagonal chambers constructed of a wood frame wrapped with 4 mil clear polyethylene plastic sheet [33]. Due to uneven micro-topography, the chambers sat approximately 3 cm off the ground, allowing for air circulation beneath the base of the greenhouses. The chambers acted as solar traps and increased the average ambient temperature at the soil surface by ~ 2.5° C. Chambers increased night temperatures on average 25% more than they increased day temperatures. In warming control plots, we installed ‘sham’ greenhouse chambers with squares cut out of each plastic panel, reducing the cover of plastic sheeting by 85%. The ambient temperature in ‘sham’ plots did not differ significantly from the ambient temperature of nitrogen control plots (no sham). Average daytime light at the soil surface did not differ between sham and non sham greenhouse chambers across the growing season. The plastic was replaced annually on all greenhouses.

We used the perennial forb *Pityopsis aspera* (Pineland silkgrass; Asteraceae) to simulate invasion in our study. *P*. *aspera* was used as an experimental invader because it occurs in the understory of surrounding long-leaf pine forests (but not in the old-field) and, therefore, could be reasonably expected to colonize the site through range filling as a response to a changing climate (e.g., [34]). *P*. *aspera* reproduces both sexually and asexually, can grow to large sizes very quickly and is pollinated by a variety of insects. We transplanted 20 adult (> 1 year old) individuals of *P*. *aspera* from existing populations in Tall Timbers into each of the invasion treatment plots. Twenty holes were excavated and refilled in plots that did not receive transplants to simulate disturbance due to transplanting.

Data were collected on biotic and abiotic factors that may be susceptible to elevated nitrogen, temperature, and invasion. These included soil characteristics, such as water-holding capacity, pH, percentage organic matter, and soil moisture; and vegetation characteristics, such as species diversity, percent invasive species, availability of understory light, foliar water content, dominant plant height, foliar toughness, and percent leaf damage from herbivores and pathogens.

### Soil characteristics

In August 2012, two sets of samples were collected from the top 7 cm of the soil profile from four random locations in each plot to determine soil moisture and soil organic matter using standard methods [35]. For both percent soil moisture and organic matter, the four measurements from each plot were averaged. In each plot, another set of soil cores 10 cm in depth were taken from four random locations, and soil pH of each core was measured using a Fieldscout SoilStick pH meter (Spectrum Technologies, Inc.). Again, these four measurements were averaged to provide a single soil pH value per plot for analysis. Single measurements of these soil characteristics were collected because we were interested in the relative differences among plots, and not necessarily absolute values of the measurements. Because, in general, soil characteristics maintain relative differences through time (e.g., [35]), we were confident that a single measurement would be reflective of soil differences among plots through the duration of the experiment.

The final abiotic characteristic measured was photosynthetically active light (PAR). PAR was estimated by measuring spectral irradiance using a LI-COR (LI-190) quantum PAR sensor at three random points on the soil surface between 1100–1300 hours EST on a cloudless day in August in 2012. These measurements were averaged within a plot to obtain one value of understory light per plot.

### Vegetation characteristics

Percent cover by species was estimated visually in each plot at peak biomass (in August of 2011 and 2012). These data were used to determine vegetation cover, species richness (number of unique species), and percent invasive species (not including *P*. *aspera)*. Functional richness was also quantified from the census data. Species were categorized into functional groups based on growth forms: native and invasive grass; native and invasive forb; legume; shrub; vine.

Some destructive sampling was necessary to quantify how vegetation was affected by the experimental treatments. As a standard, we measured several characteristics of a dominant plant *Trichostema dichotomum* (Laminaceae), an annual herb that naturally occurred in high abundances across all plots. The amount of water in the vascular tissue of *T*. *dichotomum* was determined as cellular relative water content (RWC) using modified rapid estimate procedures [36]. In June of 2012, three leaves were collected at random from *T*. *dichotomum* individuals in each plot and were weighed to determine a fresh weight (FW). Samples were then immersed in deionized water for 3 hours in a dark fridge and reweighed to determine turgid weight (TW). Finally, the samples were placed in an oven at 85°C for 24 hours and then reweighed for dry weight (DW). RWC was then found by dividing TW-DW from FW-DW.

As a proxy for biomass, we also measured the height of the four tallest individuals of *T*. *dichotomum* in each plot in both July 2011 and 2012 as the height of this species in non-experimental plots showed a strong relationship to biomass (r^2^ = 0.83, *p* < 0.001, n = 15; S1 Fig). We used these measurements to determine an average biomass per plot. A penetrometer was used to measure leaf toughness by calculating the weight necessary to puncture a hole through a leaf [37].

We also assessed herbivory and pathogen damage on *T*. *dichotomum* individuals across plots. The fourth leaf from the top of an individual *T*. *dichotomum* was collected from four individuals in each plot. Percent herbivore damage was assessed by determining how much of the total leaf area was missing due to herbivory (percent tissue removed). Percentage pathogen damage was estimated by determining how much of the total leaf area was discolored by brown lesions [38].

### Analysis

To reduce the data on species abundances to a form that was more amenable for structural equation modeling, we used NMDS ordination analyses on the 15 most common species [39], each of which was ≥ 1% proportion of the species found across the plots. We used the NMDS axes 1 and 2 (two dimensions were chosen by interpreting a scree plot of dimensionality vs. stress) to characterize the plant communities in each treatment group: the nitrogen plots (nitrogen only, nitrogen + invasion, and control) and the warming plots (warming only, warming + invasion, and control). We used a Bray-Curtis dissimilarity measure with the vegan package in R (using metaMDS) for our analysis. For each treatment group we conducted regression analysis between the NMDS scores and the percent cover of the 15 species used in the ordination to make biological interpretations of scores.

We first conducted univariate analyses to examine main and interactive effects of nitrogen addition, warming and invasion on NMDS axes scores using ANOVA. We then used structural equation modeling (SEM; using AMOS version 5.0.1; [40]) to represent and investigate the relationships among treatments, environmental response variables and the NMDS results [41]. This approach allowed us to identify plausible causal pathways among variables to understand the mechanisms driving changes in the native plant community in response to experimental treatments.

Since including all 18 measured environmental variables into SE models is not a parsimonious approach, we first wanted to identify the subset of environmental factors that most likely played a role in explaining treatment effects on the resident plant community. To do this, we overlaid data from all measured environmental variables as vectors onto the NMDS ordination to identify measured variables that had strong correlations with one or both axes (S1 Table). Environmental variables that demonstrated significant correlations with NMDS axes were then included in SEM development. For this reason, our application of SEM can be considered to be partially model-building instead of strictly hypothesis testing.

We first developed models that represented how the experimental treatments affected the plant community, represented by ordination axis scores. For the nitrogen treatment, an SEM was developed to represent a hypothesized network of interactions between the nitrogen treatment and environmental variables that could ultimately affect the native plant community in the absence of invasion. To build this model, we only used the subset of the data that did not include the invasion treatment (nitrogen only and control). A second SE model was then developed to represent how the presence of invasion might modify the network between the nitrogen treatment and environmental variables, using the subset of the data that included the invasion treatment in the presence and absence of the nitrogen treatment. We used a systematic approach (relaxing parameter constraints, one at a time) to locate inequalities between groups by examining residuals of path coefficients [42]. The indices we used to find the best overall models included a Chi-squared discrepancy function, which indicates the similarity between the observed and predicted covariance matrices (lower is better); and two goodness of fit measures, the Akaike Information Criterion (AIC), and the root mean square error of approximation (RMSEA). A *p* value indicating the significance of the Chi-squared discrepancy function (which represents a likelihood-ratio test) was also identified for each model. This value indicates the similarity between observed and predicted covariance matrices. We used the same approaches described above to develop SEMs with data from the warming treatment plots.

We also wanted to detect if and how the presence of invasion affects the network of interactions among environmental variables that drive responses of the plant community to nitrogen addition and warming. To do this, we used multigroup modeling to directly compare models in the absence and presence of invasion (models compared were nitrogen only vs. nitrogen + invasion models, and warming only vs. warming + invasion). We first conducted an invariance (similarity) test which compared the comparative fit index (ΔCFI; [43]) between the configural model (no equality constraints imposed) and the measurement model (all equality constraints imposed). If invariance was found, we proceeded to sequentially modify equality constraints on each factor so that we could assess invariance among models where parameters were freely estimated using a ΔCFI cutoff of 0.01 [44].

## Results and Discussion

The resident community contained over 53 species; some of the more abundant species included a flatsedge, *Cyperus stigosus*, along with the annual herbs *Ambrosia artemisiifolia* and *Trichostema dichotomum*, the perennial herbs *Polypremum procumbens*, *Rhexia maiana*, and *Campsis radicans*, and two small shrubs, *Rubus flagellaris* and *R*. *cuneifolius*. The species varied in their responses to the treatments. The addition of *Pityopsis aspera*, for example, seemed to increase the abundance of *T*. *dichotomum* and *Cyperus stigosus*, but decreased other species, such as *R*. *flagellaris* and *Campsis radicans* (S2 Fig). Nitrogen addition plots had almost 70% more *P*. *procumbens* than control plots, but fewer individuals of *Helianthus angustifolius* and *Monarda punctata*. The warming treatment was correlated with slight increases in many species, but especially *Polypremum procumbens*, *A*. *artemisifolia*, and *T*. *dichotomum*. Other species showed decreases to the warming, including *S*. *canadensis* and *R*. *flagellaris*. Although there was no significant difference in species richness across plots, nitrogen plots were comprised of a smaller number of invasive annuals (*p* = 0.003) and a larger number of native climbing plants (*p* = 0.03).

Percent damage by herbivory and pathogens and soil moisture were lowest in the nitrogen addition plots. Conversely, soil pH was highest in the nitrogen plots (Table 1). This was not entirely unexpected as nitrogen application has been shown to increase pH through increased above- and below-ground plant growth, which results in excess nitrate capture, biocarbonate release and the ultimate reduction of soil acidity [45]. In the warming plots, leaf toughness was lowest and demonstrated the least variation compared to plots exposed to all other treatments. Percent soil organic material was also highest in these plots (Table 2). The ANOVA results suggest that NMDS1 scores were most affected by the interaction between warming and invasion (*p* = 0.07), compared to all other treatment effects. The NMDS2 scores were affected by nitrogen addition (*p* = 0.05), warming (*p* = 0.05), and warming + invasion (*p* = 0.01).

**Table 1:** Means and (SD) for environmental factors from plots exposed to the nitrogen treatment (data from warming treatment plots excluded).

	No nitrogen added		Nitrogen added	
	*Invasion -*	*Invasion +*	*Invasion -*	*Invasion +*
Species richness 2011*	9.4 (3.0)	11.4 (2.6)	11.4 (4.5)	12.2 (3.1)
Species richness 2012*	9.6 (2.9)	9.7 (2.4)	10.4 (2.3)	9.2 (2.2)
% Invasive 2011	16.5 (9.2)	7.4 (6.5)	7.8 (4.6)	11.2 (1.7)
% Invasive 2012*	17.0 (8.7)	5.9 (5.3)	10.1 (2.6)	14.0 (9.2)
Functional richness	4.5 (0.7)	4.5 (0.8)	4.7 (0.5)	4.6 (0.7)
Foliar water content	0.1 (0.0)	0.1 (0.1)	0.2 (0.3)	0.2 (0.3)
Biomass 2011	100.1 (13.7)	96.6 (15.2)	96.5 (18.8)	97.2 (17.7)
Biomass 2012	74.6 (14.7)	75.9 (10.5)	86.0 (13.6)	78.2 (13.7)
Foliar toughness	87.3 (33.1)	92.7 (53)	73.8 (26.8)	77.3 (32.0)
Soil pH*	5.1 (0.4)	5.4 (0.2)	7.2 (3.1)	5.6 (0.4)
Soil moisture*	9.2 (1.0)	9.0 (0.7)	8.8 (1.5)	9.2 (2.2)
% Soil organic	3.8 (2.7)	3.0 (0.7)	2.9 (0.3)	3.0 (0.5)
Water holding capacity	27.5 (1.2)	27.8 (1.4)	27.3 (2.3)	34.2 (13.3)
Solar radiation	109.9 (87.8)	158.1 (120.7)	83.8 (65.9)	84.8 (59.2)
% Herbivory	4.6 (4.6)	3.5 (4.3)	6.0 (9.6)	0.0 (0.0)
% Pathogen	3.0 (2.6)	2.8 (3.2)	8.0 (12.6)	4 (3.5)

Factor names followed by an * were included in the structural equation models for nitrogen plots.

**Table 2:** Means and (SD) for environmental factors from plots exposed to the warming treatment (data from nitrogen addition plots excluded).

	No warming		Warming	
	*Invasion -*	*Invasion +*	*Invasion -*	*Invasion +*
Species richness 2011	8.6 (2.9)	12.6 (2.3)	11 (2.9)	12.6 (2.1)
Species richness 2012*	9.6 (2.7)	8.8 (2.5)	8.6 (1.3)	10.0 (3.1)
% Invasive 2011*	15.4 (8.1)	4.5 (6.3)	11.7 (9.1)	11.2 (4.3)
% Invasive 2012	13.3 (14.7)	6.5 (6.1)	7.5 (16.8)	19.4 (13.5)
Functional richness	4.4 (0.5)	4.2 (0.8)	4.4 (0.8)	4.6 (0.7)
Foliar water content	0.1 (0.0)	0.2 (0.1)	0.1 (0.0)	0.1 (0.0)
Biomass 2011	97.0 (16.8)	97.0 (6.7)	95.2 (7.7)	101.5 (13.1)
Biomass 2012	72.8 (6.9)	81.3 (8.3)	90.2 (11.6)	93.6 (17.4)
Foliar toughness	94.1 (43.4)	112.4 (70.0)	61.5 (20.8)	69.9 (20.8)
Soil pH*	5.2 (0.1)	5.5 (0.2)	5.2 (0.4)	5.5 (0.7)
Soil moisture	9.6 (0.7)	8.8 (0.6)	9.5 (0.4)	8.7 (0.7)
% Soil organic	2.9 (0.7)	3.0 (0.5)	3.2 (0.4)	3.4 (0.5)
Water holding capacity	27.3 (1.1)	27.7 (1.1)	27.4 (1.7)	27.1 (0.7)
Solar radiation	71.6 (78.1)	176.0 (140.5)	120.5 (97.0)	71.9 (44.8)
% Herbivory*	4.2 (5.8)	5.8 (4.8)	12.5 (25)	3.6 (3.3)
% Pathogen*	3.0 (2.5)	4.2 (4.2)	5.8 (5.9)	3.6 (2.3)

Factor names followed by an * were included in the structural equation models for warming plots.

### Nitrogen addition

The metaMDS analysis suggests that our NMDS results were relatively robust (stress = 0.23; R^2^ = 0.92). Higher values of NMDS1 were correlated with increasing cover of the dominant *T*. *dichotomum* (*p* = 0.03) and the invasive *P*. *notatum* (*p* = 0.02) and decreasing cover of the relatively rare species *H*. *angustifolius* (p = 0.02), and *R*. *cuneifolius* (*p* < 0.0001). Alternatively, higher values of NMDS2 reflected increasing cover of *M*. *punctata* (*p* = 0.01), and the dominant graminoid *C*. *strigosus* (*p* = 0.008). Overall, functional richness was negatively correlated with NMDS1 (*p* = 0.02) and positively correlated with NMDS2 (*p* = 0.03).

Five predictor variables were included in the first SE model (constructed using nitrogen addition and control data) because of correlations with NMDS axes (S2 Table), including species richness in 2011 (r^2^ = 0.483, *p* = 0.002) and 2012 (r^2^ = 0.583, *p* = 0.001); percentage invasive plants per plot in 2012 (r^2^ = 0.216, *p* = 0.08), soil pH (r^2^ = 0.225, *p* = 0.07), and soil moisture (r^2^ = 0.212, *p* = 0.07). Because percent invasive plants per plot in 2012 was strongly correlated with species richness in 2012 (S2 Table), the percent invasive variable was excluded from model construction.

**Fig 1:**
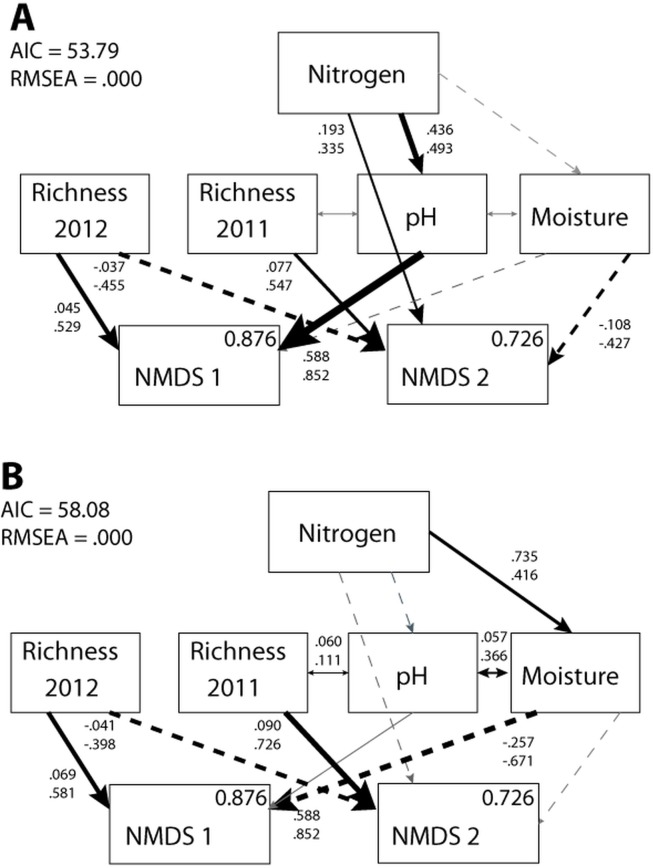
Results of the nitrogen SEM. Nitrogen effects on plant community NMDS scores in the (A) absence (χ² = 1.79, d.f. = 9, *p* = 0.994) and (B) presence of invasion (χ² = 6.08, d.f. = 9, *p* = 0.732). Black arrows with values indicate significant paths, gray arrows indicate insignificant paths, solid arrows indicate positive relationships, and dashed arrows indicate negative relationships. Path values indicate unstandardized (top) and standardized (bottom) coefficients. Within box values indicate R^2^ values, which represent the proportion of variance explained for each response variable.

In the absence of invasion, the most parsimonious model predicting nitrogen effects demonstrated that species richness in 2011 and 2012 had opposing relationships with the native plant community (Fig 1A). There was a positive relationship between species richness in 2011 and NMDS2 scores (increased functional richness and cover by a dominant graminoid), but these community values had a negative relationship with species richness in 2012. Elevated soil pH increased functional species richness (Table 1), possibly explained by changes in species-specific plant growth capabilities (e.g., [46, 47, 48]). The subsequent change in NMDS1 scores was likely due to disproportionate benefit of dominant and invasive species to the modification of soil pH [49]. Nitrogen addition likely affected unmeasured plant traits related to competition, such as the growth rate or stomatal conductance of rare species, subsequently modifying the dominance structure of the community. Finally, the direct path from nitrogen to NMDS2 indicates that a portion of the nitrogen treatment effect on changes in the native plant community was not accounted for by any of the environmental variables present in the model (Fig 1A).

In general, paths in the second SEM (including invasion only and nitrogen + invasion) were similar in direction (but not in magnitude) to those found in the nitrogen only model (Fig 1B). In the presence of invasion, species richness in 2011 and 2012 had opposite correlations with the native plant community in 2012. Differences between models did exist, however. In the presence of invasion, soil pH no longer explained changes in community composition. *P*. *aspera* could have amended soil nitrogen availability or fixation rates [50], obfuscating nitrogen effects on soil acidity (e.g., [51]), and explaining the loss of the role of soil pH in the model. Additionally, in the presence of invasion, nitrogen indirectly affected the native plant community through changes in soil moisture (Fig 1B), instead of through changes in soil pH (ΔCFI = 0.163). This increase in soil moisture could have resulted from the experimental invasion if *P*. *aspera* supplied adequate shade to reduce moisture loss at the soil surface through its living tissue [52], or through litter deposition [53].

### Warming

Two dimensions of the NMDS (stress = 0.23) resulted in a non-metric R^2^ = 0.94. Higher values of NMDS1 were correlated with increases in the cover of common (but not dominant) species: *C*. *radicans* (*p* = 0.009), *R*. *flagellaris* (*p* < 0.001), and *S*. *auriculata* (*p* = 0.05), in addition to the dominant graminoid *Cyperus strigosus* (*p* = 0.01). Higher values of NMDS1 were also correlated with decreases in the cover of the exotic graminoid *Paspalum urvillei* (*p* = 0.004). Alternatively, higher values of NMDS2 were correlated with increases in the cover of the common shrub *R*. *cuneifolius* (*p* = 0.001), and with decreases in the cover of the common *Campsis radicans* (*p* = 0.008), and *R*. *flagellaris* (*p* = 0.02). Overall, functional richness was positively correlated with NMDS1 (*p* = 0.08), but demonstrated no relationship with NMDS2.

The predictor variables included in the first SEM (constructed using warming and control data) because of significant correlations with NMDS axes (S2 Table), included expected changes in biodiversity [54]: species richness in 2011 (r^2^ = 0.254, *p* = 0.04), species richness in 2012 (r^2^ = 0.238, *p* = 0.06) percent invasive species in 2011 (r^2^ = 0.215, *p* = 0.06), likely as a result of a non-random reduction in the abundance of species with traits that are particularly vulnerable to changes in temperature [55, 56]. Other predictor variables included foliar herbivory damage (r^2^ = 0.217, *p* = 0.06), foliar pathogen damage (r^2^ = 0.177, *p* = 0.09), and soil pH (r^2^ = 0.315, *p* = 0.01). These changes in pathogen and herbivory loads can be important drivers of productivity and composition across resource and environmental gradients [57]. Because species richness in 2011 was strongly correlated with species richness in 2012 and percent invasive species in 2011 (S2 Table), this variable was excluded from model construction.

**Fig 2:**
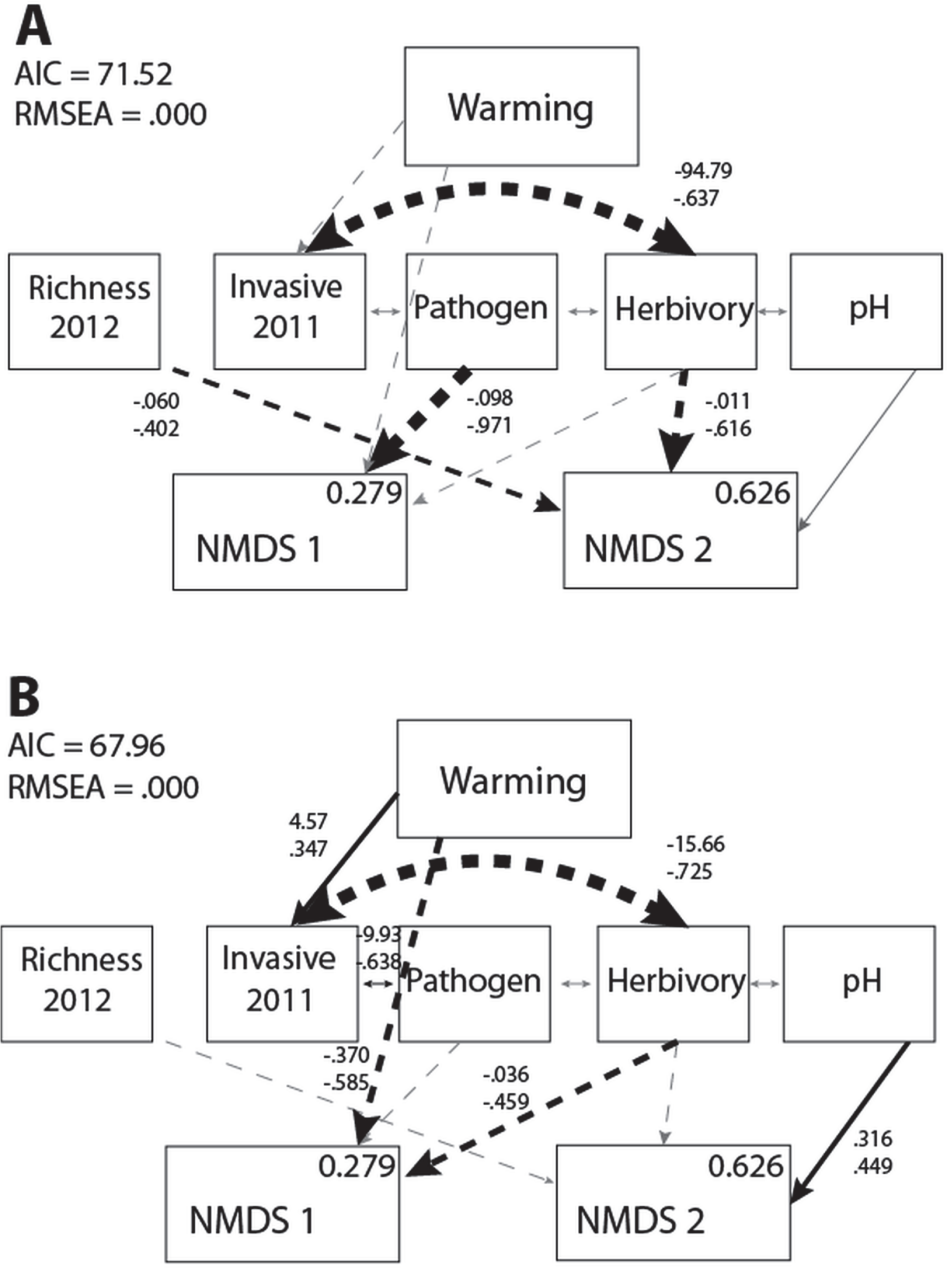
Results of the warming SEM. Warming effects on plant community NMDS scores in the (A) absence (χ² = 15.519, d.f. = 16, *p* = 0.487) and (B) presence of invasion (χ² = 10.002, d.f. = 16, *p* = 0.867). Arrow characteristics and all values as in Fig 1.

In the absence of invasion, the most parsimonious model predicting warming effects demonstrated that increased pathogen damage was negatively correlated with functional richness, potentially through an increase in exotic species as NMDS1 scores decrease (Fig 2A). Species richness in 2012 and herbivory acted in concert to reduce NMDS2 scores, which was characterized by a reduction in dominant cover across plots. Finally, the effects of percent invasive species in 2011 and pH modified the native plant community, but only indirectly through other environmental factors such as herbivory and pathogen damage.

The presence of the invasion treatment significantly modified the way in which warming affected the native plant community (ΔCFI = 0.141). In the presence of invasion, warming directly decreased functional richness and increased the cover of exotics, while indirectly affecting NMDS1 scores through invasion and herbivory (Fig 2B). Invasive species have been shown to facilitate further invasion [58], and, although this is also expected in the presence of warming [59], the explanatory role of invasive species in 2011 occurs with an intermediary pathway to herbivore damage. Warming served to reduce herbivory on a dominant plant, perhaps because individuals experiencing herbivory responded to elevated temperatures by shortening their life cycle [60], which subsequently reduced foliar damage [61]. The presence of the invasive *P*. *aspera* could have served to modify the presence of herbivory indirectly, for example, by facilitating differences in plant defenses or soil characteristics, or directly by reducing herbivore presence [62]. Warming could have encouraged this response as well [63], but perhaps this signal of herbivore damage could only be identified when the effect was exacerbated by the presence of a novel invasive species.

Invasion also modified direct paths from environmental factors to NMDS scores. For example, although species richness in 2012 and pathogen damage no longer played a direct role in explaining changes in the native plant community, decreasing acidity (increasing pH values) appeared to encourage the cover of the graminoid dominant *C*. *strigosus*. Increasing damage from herbivory continued to have a significant effect on the native plant community, but, in the presence of invasion, resulted in a decrease in both functional richness and the cover of some common species (Table 2).

## Conclusion

Combined effects and simultaneous responses of climate change are common and persistent across systems. Although resident plants and habitats are vulnerable to effects of climate change and non-aggressive invaders, empirical investigations to understand the collective behavior of these factors are almost nonexistent [64]. We found that, over a short time scale, elevated temperature and nitrogen deposition altered the resident plant community across experimental plots. Both the magnitude, and often, the sign of the paths among treatments, biotic and abiotic variables and plant community ordination scores differed in the presence and absence of invasion. This work illustrates how relationships among factors of climate change and invasion can be complex and non-additive. Moreover, to appreciate how climate change can modify native plants, it is not adequate to merely document final community effects. Future research should focus on identifying the identity and the magnitude to which biotic and abiotic variables are responsible for explaining relationships among climate change factors and plant communities.

## Supporting Information

S1 Fig Relationship between main stem height and adult biomass for *Trichostema dichotomum*. (DOCX)

S2 Fig Mean ± SD for the 15 most abundant plant species found across plots. (A) control, (B) nitrogen, and (C) warming treatments in the absence (white bars) and presence (black bars) of invasion. Species are arranged in functional groups. Annual herbs: AMAR = *Ambrosia artemisiifolia*, TRDI = *Trichostema dichotomum*. Perennial herbs: SOCA = *Solidago canadensis*, HEAU = *Helianthus austifolia*, MOPU = *Monarda punctate*, POPR = *Polypremum procumbens*, DICA = *Dichondra carolinensis*, RHMA = *Rhexia maiana*; Vines: CARA = *Campsis radicans*, SMAU = *Smilax auriculata*; Shrubs: RUCU = *Rubus cuneifolius*, RUFL = *Rubus flagellaris*; and Graminoids: PAUR = *Paspalum urvillei*, CYST *= Cyperus strigosus*, PANO = *Paspalum notatum*.
(DOCX)

S1 Table NMDS scores and correlations of measured environmental variables and ordination axes. (DOCX)

S2 Table Scatterplot of relationships among measured environmental variables. (DOCX)
