# Supplementary material for: Plant Community Responses to Simultaneous Changes in Temperature, Nitrogen Availability, and Invasion
Source: PLoS One. 2015 Apr 16;10(4):e0123715. doi: 10.1371/journal.pone.0123715 (PMC4400009; doi:10.1371/journal.pone.0123715)

**Supporting Information Figure S1**. Relationship between main stem height and adult biomass for *Trichostema dichotomum*. Stems of 15 individuals were measured in the field, and then brought to the lab. The samples were dried in an oven at 60 °C for 24 hours and weighed to determine biomass.


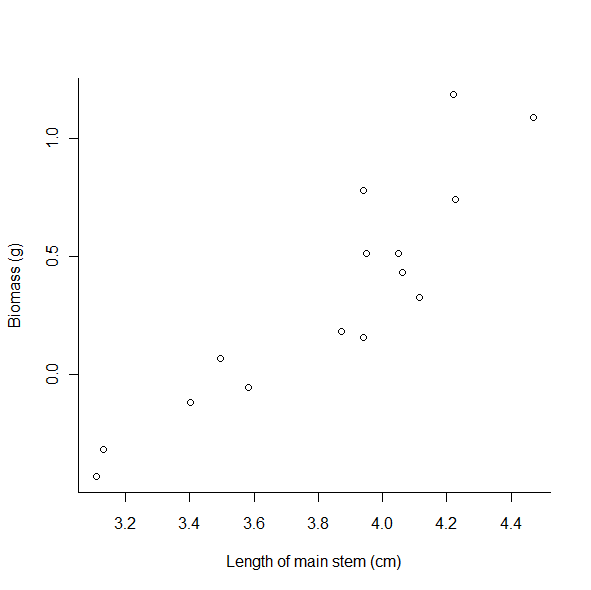

Supplement: S1 Fig — (DOCX) [file pone.0123715.s001.docx]
