# Supplementary material for: Plant Community Responses to Simultaneous Changes in Temperature, Nitrogen Availability, and Invasion
Source: PLoS One. 2015 Apr 16;10(4):e0123715. doi: 10.1371/journal.pone.0123715 (PMC4400009; doi:10.1371/journal.pone.0123715)
Supplement: S2 Fig — (A) control, (B) nitrogen, and (C) warming treatments in the absence (white bars) and presence (black bars) of invasion. Species are arranged in functional groups. Annual herbs: AMAR = Ambrosia artemisiifolia, TRDI = Trichostema dichotomum. Perennial herbs: SOCA = Solidago canadensis, HEAU = Helianthus austifolia, MOPU = Monarda punctate, POPR = Polypremum procumbens, DICA = Dichondra carolinensis, RHMA = Rhexia maiana; Vines: CARA = Campsis radicans, SMAU = Smilax auriculata; Shrubs: RUCU = Rubus cuneifolius, RUFL = Rubus flagellaris; and Graminoids: PAUR = Paspalum urvillei, CYST = Cyperus strigosus, PANO = Paspalum notatum. (DOCX) [file pone.0123715.s002.docx]

**Supplementary Information Figure S2**. **Mean ± SD for the 15 most abundant plant species found across plots.** (A) control, (B) nitrogen, and (C) warming treatments in the absence (white bars) and presence (black bars) of invasion. Species are arranged in functional groups. Annual herbs: AMAR = *Ambrosia artemisiifolia*, TRDI = *Trichostema dichotomum*. Perennial herbs: SOCA = *Solidago canadensis*, HEAU = *Helianthus austifolia*, MOPU = *Monarda punctate*, POPR = *Polypremum procumbens*, DICA = *Dichondra carolinensis*, RHMA = *Rhexia maiana*; Vines: CARA = *Campsis radicans*, SMAU = *Smilax auriculata*; Shrubs: RUCU = *Rubus cuneifolius*, RUFL = *Rubus flagellaris*; and Graminoids: PAUR = *Paspalum urvillei*, CYST *= Cyperus strigosus*, PANO = *Paspalum notatum*.

| 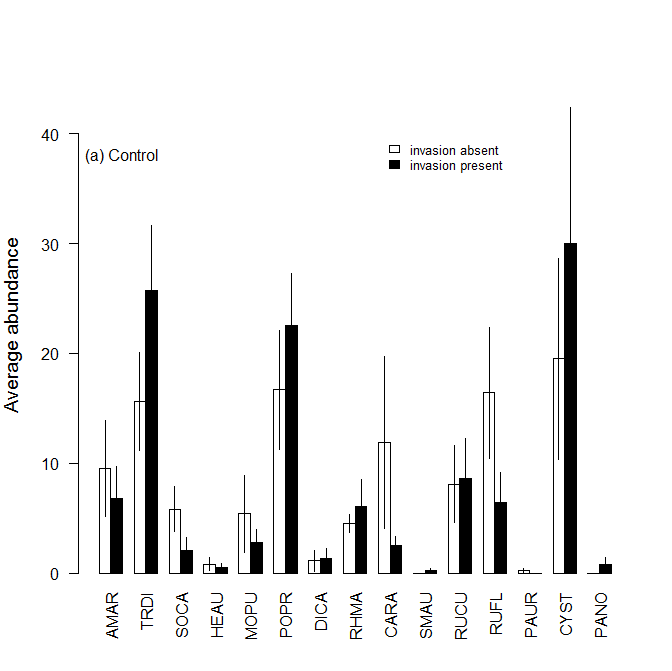 |
| --- |
| 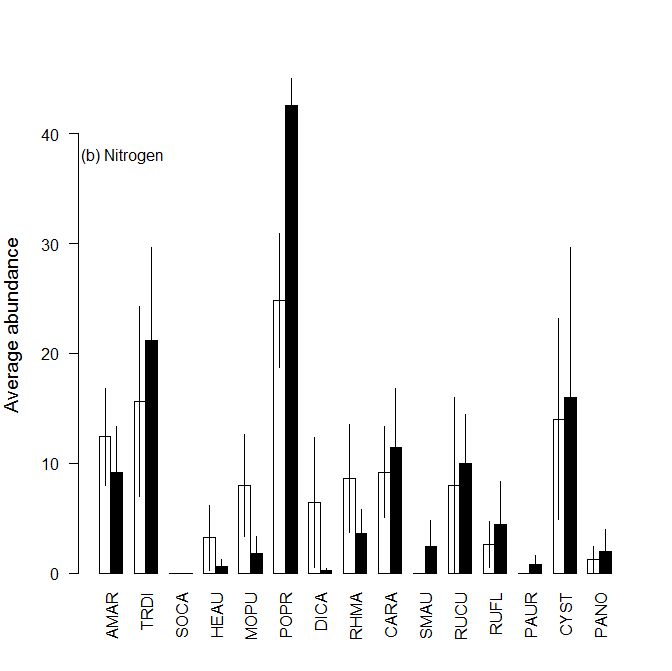 |
| 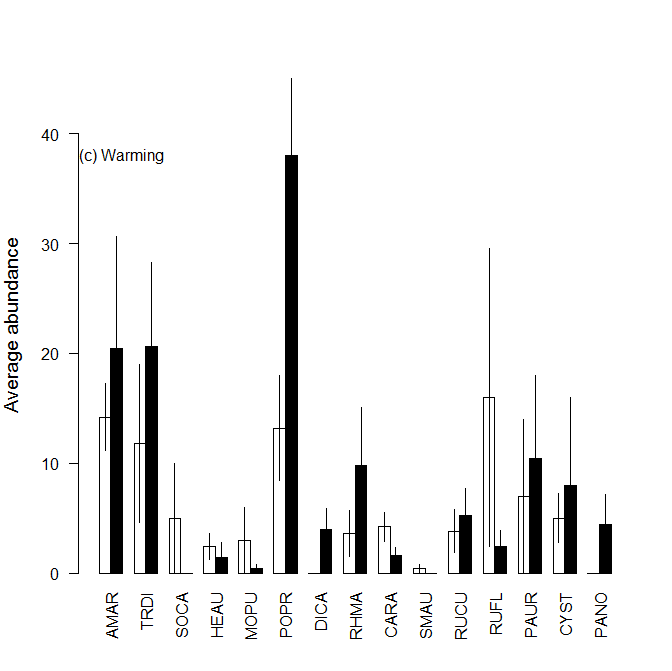 |
