# Supplementary material for: Plant Community Responses to Simultaneous Changes in Temperature, Nitrogen Availability, and Invasion
Source: PLoS One. 2015 Apr 16;10(4):e0123715. doi: 10.1371/journal.pone.0123715 (PMC4400009; doi:10.1371/journal.pone.0123715)
Supplement: S1 Table — (DOCX) [file pone.0123715.s003.docx]

**Supporting Information Table S1**. NMDS scores and correlations of measured environmental variables and ordination axes for plots exposed to the nitrogen treatment.

|  | **NMDS1** | **NMDS2** | **r^2^** | **P** |
| --- | --- | --- | --- | --- |
| Richness 2011 | 0.915 | -0.403 | 0.483 | 0.002 |
| Richness 2012 | 0.412 | 0.911 | 0.583 | 0.001 |
| Invasive 2011 | -0.972 | -0.237 | 0.029 | 0.731 |
| Invasive 2012 | -0.072 | -0.997 | 0.216 | 0.083 |
| Biomass 2011 | -0.255 | 0.997 | 0.025 | 0.781 |
| Biomass 2012 | -0.916 | 0.401 | 0.005 | 0.953 |
| Water holding capacity | 0.980 | 0.199 | 0.053 | 0.679 |
| Relative water content | 0.993 | 0.119 | 0.101 | 0.358 |
| Leaf toughness | 0.940 | 0.340 | 0.192 | 0.108 |
| Herbivory | -0.837 | 0.548 | 0.061 | 0.564 |
| Pathogen | 0.106 | 0.994 | 0.061 | 0.564 |
| Soil pH | 0.847 | 0.532 | 0.225 | 0.076 |
| Soil moisture | -0.807 | -0.590 | 0.212 | 0.074 |
| Soil organic | -0.787 | -0.617 | 0.174 | 0.146 |
| Solar radiation | 0.999 | -0.031 | 0.159 | 0.176 |

NMDS scores and correlations of measured environmental variables and ordination axes for plots exposed to the warming treatment.

|  | **NMDS1** | **NMDS2** | **r^2^** | **P** |
| --- | --- | --- | --- | --- |
| Richness 2011 | -0.999 | -0.038 | 0.254 | 0.043 |
| Richness 2012 | -0.850 | -0.526 | 0.238 | 0.058 |
| Invasive 2011 | 0.999 | -0.043 | 0.215 | 0.061 |
| Invasive 2012 | -0.030 | 0.999 | 0.151 | 0.154 |
| Biomass 2011 | -0.462 | 0.887 | 0.064 | 0.491 |
| Biomass 2012 | -0.714 | -0.699 | 0.032 | 0.684 |
| Water holding capacity | 0.444 | 0.896 | 0.038 | 0.649 |
| Relative water content | -0.998 | -0.057 | 0.010 | 0.886 |
| Leaf toughness | 0.313 | 0.950 | 0.056 | 0.507 |
| Herbivory | -0.230 | -0.973 | 0.217 | 0.065 |
| Pathogen | - 0.847 | -0.532 | 0.177 | 0.091 |
| Soil pH | -0.173 | 0.985 | 0.315 | 0.015 |
| Soil moisture | 0.822 | 0.569 | 0.114 | 0.244 |
| Soil organic | -0.210 | 0.978 | 0.073 | 0.407 |
| Solar radiation | -0.767 | -0.642 | 0.041 | 0.600 |
