# Supplementary material for: Plant Community Responses to Simultaneous Changes in Temperature, Nitrogen Availability, and Invasion
Source: PLoS One. 2015 Apr 16;10(4):e0123715. doi: 10.1371/journal.pone.0123715 (PMC4400009; doi:10.1371/journal.pone.0123715)

**Supporting Information Table S2.** Scatterplot of relationships among measured environmental variables in the nitrogen plots.


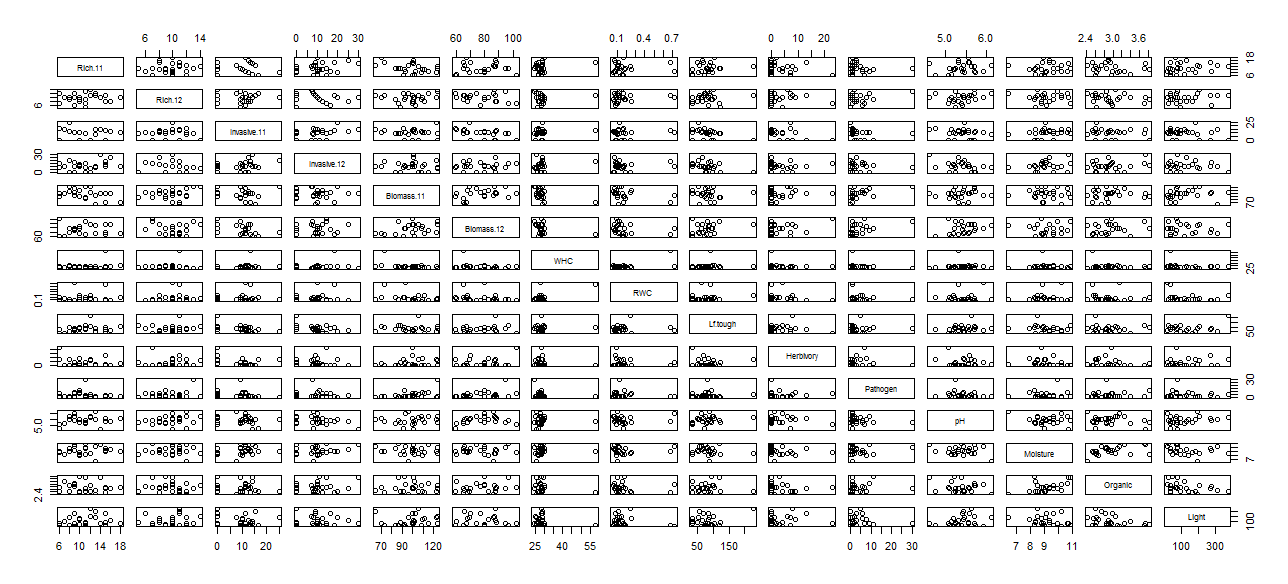


Scatterplot of relationships among measured environmental variables in the warming plots.


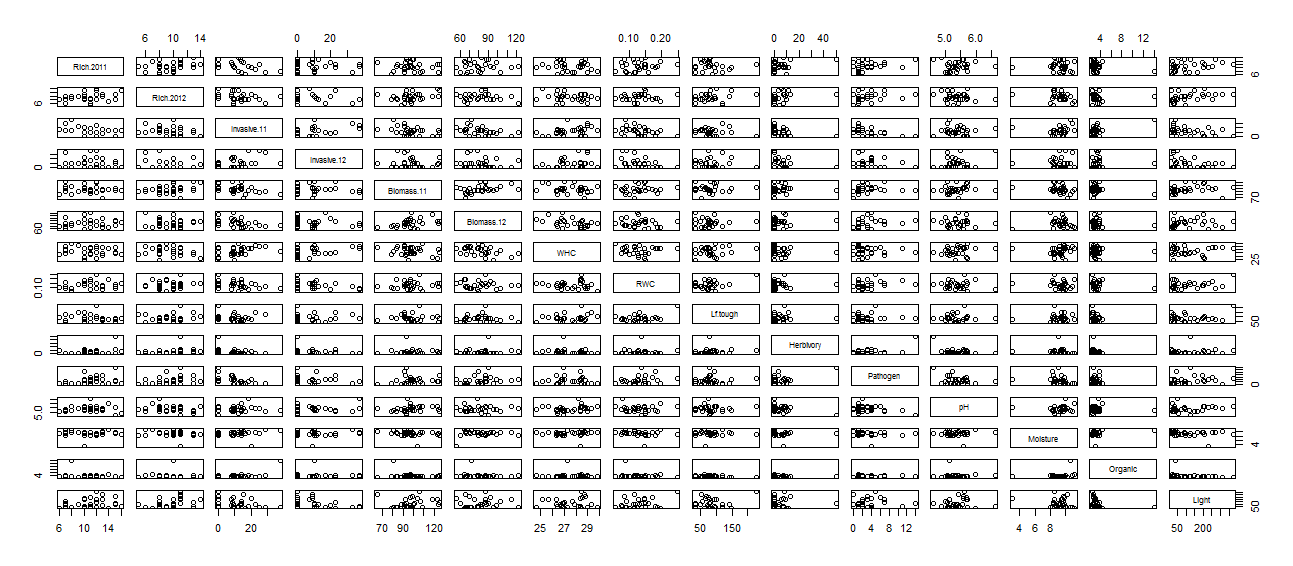

Supplement: S2 Table — (DOCX) [file pone.0123715.s004.docx]
